# Effects of Supplementation of Branched-Chain Amino Acids to Reduced-Protein Diet on Skeletal Muscle Protein Synthesis and Degradation in the Fed and Fasted States in a Piglet Model

**DOI:** 10.3390/nu9010017

**Published:** 2016-12-28

**Authors:** Liufeng Zheng, Hongkui Wei, Pingli He, Shengjun Zhao, Quanhang Xiang, Jiaman Pang, Jian Peng

**Affiliations:** 1 Department of Animal Nutrition and Feed Science, College of Animal Science and Technology, Huazhong Agricultural University, Wuhan 430070, China; zhenglf2013@webmail.hzau.edu.cn (L.Z.);weihongkui@mail.hzau.edu.cn (H.W.); xiangquanhang@webmail.hzau.edu.cn (Q.X.); pjm@webmail.hzau.edu.cn (J.P.); 2 The Cooperative Innovation Center for Sustainable Pig Production, Wuhan 430070, China; 3 State Key Laboratory of Animal Nutrition, College of Animal Science and Technology, China Agricultural University, Beijing 100094, China; hepingli@cau.edu.cn; 4 Department of Feed Science, Wuhan Polytechnic University, Wuhan 430023, China; zhaoShengjun1974@163.com

**Keywords:** piglets, branched-chain amino acids, muscle protein synthesis, muscle protein degradation, α-ketoisocaproate

## Abstract

Supplementation of branched-chain amino acids (BCAA) has been demonstrated to promote skeletal muscle mass gain, but the mechanisms underlying this observation are still unknown. Since the regulation of muscle mass depends on a dynamic equilibrium (fasted losses–fed gains) in protein turnover, the aim of this study was to investigate the effects of BCAA supplementation on muscle protein synthesis and degradation in fed/fasted states and the related mechanisms. Fourteen 26- (Experiment 1) and 28-day-old (Experiment 2) piglets were fed reduced-protein diets without or with supplemental BCAA. After a four-week acclimation period, skeletal muscle mass and components of anabolic and catabolic signaling in muscle samples after overnight fasting were determined in Experiment 1. Pigs in Experiment 2 were implanted with carotid arterial, jugular venous, femoral arterial and venous catheters, and fed once hourly along with the intravenous infusion of NaH^13^CO_3_ for 2 h, followed by a 6-h infusion of [1-^13^C]leucine. Muscle leucine kinetics were measured using arteriovenous difference technique. The mass of most muscles was increased by BCAA supplementation. During feeding, BCAA supplementation increased leucine uptake, protein synthesis, protein degradation and net transamination. The greater increase in protein synthesis than in protein degradation resulted in elevated protein deposition. Protein synthesis was strongly and positively correlated with the intramuscular net production of α-ketoisocaproate (KIC) and protein degradation. Moreover, BCAA supplementation enhanced the fasted-state phosphorylation of protein translation initiation factors and inhibited the protein-degradation signaling of ubiquitin-proteasome and autophagy-lysosome systems. In conclusion, supplementation of BCAA to reduced-protein diet increases fed-state protein synthesis and inhibits fasted-state protein degradation, both of which could contribute to the elevation of skeletal muscle mass in piglets. The effect of BCAA supplementation on muscle protein synthesis is associated with the increase in protein degradation and KIC production in the fed state.

## 1. Introduction

Branched-chain amino acids (BCAA; leucine, isoleucine and valine), which serve as the signaling AA, have attracted considerable research interests in the last decade. It has been well demonstrated that BCAA, especially leucine, specifically induces the protein synthetic response to the postprandial increase of circulating AA in skeletal muscle [1,2]. Moreover, leucine has great potential to be added to reduced-protein diets with the aim to promote muscle anabolism in piglets [3,4,5]. However, a deficiency in valine and isoleucine may reduce the effect of leucine supplementation to enhance muscle protein synthesis and growth [6]. Indeed, our recent study found that the muscle mass and protein synthetic signaling can be significantly increased by dietary supplementation of all three BCAA in piglets [7].

Although our previous study suggested that skeletal muscle mass was increased by BCAA supplementation [7], the exact mechanisms underlying this observation remain largely unknown. Regulation of muscle mass depends on the dynamic equilibrium (fasted losses–fed gains) between protein synthesis and degradation [8,9]. During feeding, the rapid muscle mass gain is sustained by the marked increase in muscle protein synthesis of piglets [10,11]. Free intracellular pool of AA, especially essential AA, is the most critical determinant of protein synthesis. These AA can be derived from not only inward flux from plasma but also protein degradation [12]. In contrast to the whole body proteolysis, muscle protein degradation is not decreased, but rather is increased in piglets during feeding [11]. The important role of proteolysis in regulation of protein synthesis has been demonstrated recently [13]. Besides the supply from the artery, a continuous supply of free AA from protein degradation may also be critical for sustaining the high-rate protein synthesis. However, little is known about whether the elevation of protein degradation contributes to the BCAA-induced increase of muscle protein synthesis in the fed state.

On the other hand, in most types of muscle atrophy induced by various stimuli including fasting, the loss of mass is mainly driven by the rapid increase of protein degradation [14,15]. Intriguingly, oral administration of both leucine and α-ketoisocaproate (KIC) after 24 h fasting can suppress protein degradation and down-regulate the expression of proteolysis-related genes in skeletal muscle [16,17,18], particularly KIC [17]. Thus, it is important to determine whether the chronic intake of BCAA-supplemented diet can maintain skeletal muscle mass by suppressing proteolysis in the fasted state.

Moreover, it is notable that besides serving as building blocks for proteins, BCAA can be extensively catabolized not only within pancreas, liver and kidney, but also within skeletal muscle [19,20], which poses the possibility that BCAA metabolites are involved in mediating the anabolic effects of BCAA on muscle growth. Recently, it has been found that the administration of leucine metabolites, including KIC and β-hydroxy-β-methylbutyrate (HMB), can increase protein synthesis through activating translation initiation factors [21,22]. However, little information is available about the association between leucine catabolism and its stimulation on protein synthesis in skeletal muscle in vivo. Meanwhile, it is unclear whether the conversion of leucine to KIC is required for a potent stimulation of protein synthesis by leucine. Since skeletal muscle has a limited capacity to further produce HMB from KIC [23], it seems unlikely that HMB production contributes to the anabolic effects of leucine in skeletal muscle. Additionally, although the hindlimb flux model by implanting catheters into femoral artery and vein has been well established [24], to the best of our knowledge, there have been few reports about the application of this model to study protein and AA metabolism in the muscle of piglets.

Taken together, we hypothesized that: (1) supplementation of BCAA to reduced-protein diet stimulates muscle protein synthesis during feeding and inhibits muscle protein degradation during fasting, leading to the increase in muscle protein deposition and mass; and (2) the beneficial effect of BCAA supplementation on protein synthesis during feeding is associated with the increases in protein degradation and KIC production from leucine catabolism in the muscle. Therefore, the piglet model of arteriovenous balance across hindlimb muscle was combined with a stable isotope tracer approach to investigate the metabolic fate of leucine in hindlimb muscle and the contribution of muscle protein degradation and KIC production to muscle protein synthesis during feeding of a BCAA-supplemented diet. Moreover, components of protein synthesis and degradation signaling in skeletal muscle after an overnight fasting were detected to explore the impacts of BCAA supplementation on protein turnover under catabolic condition.

## 2. Materials and Methods

Two experiments were conducted. The protocol was approved by the Animal Care and Use Committee of College of Animal Sciences and Technology, Huazhong Agricultural University (approval permit number 30700571) and was conducted in accordance with the National Research Council’s Guide for the Care and Use of Laboratory Animals.

### 2.1. Animals and Diets

In Experiment 1, animals and diets were previously described [7]. Briefly, fourteen Large White × Landrace barrows weaned at 26 days of age were housed individually in 1.5 × 0.75 m^2^ metabolic cages located in the same air-conditioned room with a 12-h light and 12-h dark cycle, and were adapted to a commercial diet for 7 days. On the basis of the origin of litters and body weights (BW), all pigs were randomly assigned to receive either of the two experimental diets (*n* = 7/group): the reduced-crude protein (CP) diet without (control) or with (treatment) supplemental BCAA. Room temperature was maintained at 25–28 °C. Pigs had free access to feed and drinking water. The final body weights and average daily feed intake after 4-week experimental period had been shown in our previous study [7].

The two isonitrogenous and isocaloric diets were formulated to meet the NRC [25] recommended requirements of the standardized ileal digestible (SID) AA (except for BCAA) for pigs weighing 11–25 kg [7]. The ingredients of the diets and the amounts of added synthetic AA are summarized in Table 1. Both diets had the same CP content of 16.7%, which was 20% lower than that recommended by NRC [26]. Control diet was formulated without the addition of BCAA, but was supplemented with 0.42% alanine, and therefore it was deficient of valine and isoleucine (13.2% and 8.6% lower than the NRC (2012) recommendation, respectively) [25]. Considering that deficiency of valine and isoleucine limits the ability of leucine supplementation to enhance muscle protein synthesis [6], treatment diet was formulated by supplementing 0.17% isoleucine, 0.24% leucine and 0.16% valine (all of 99% purity; Amresco) to the control diet to provide SID BCAA equal to the level in the diet with protein content (20% CP) recommended by NRC [26].

One pig from the control group was excluded because of death due to acute respiratory disease. To better confirm the beneficial effect of BCAA supplementation on muscle mass of piglets in the pair-fed experiment in our previous study [7], at the end of the experiment, six pigs were selected randomly from each group to be humanely euthanized by electrical stunning, coupled with exsanguination after 12 h of feed deprivation. To obtain plasma, the blood from precaval vein was collected in heparinized tubes and centrifuged at 4 °C at 3000× *g* for 10 min. The samples were then stored at −80 °C until analysis of insulin. In total, 23 major individual skeletal muscles from the forequarter, midquarter and hindquarter of the left carcass side were separated entirely and weighed. The longissimus dorsi (LD) muscle samples between the 10th and the last rib on the right carcass side were rapidly removed, wrapped in foil and frozen in liquid N_2_ to be stored at –80 °C until protein determination. Another set of LD muscle samples were also collected from each pig and fixed in 10% formaldehyde-phosphate buffer, and kept at 4 °C for morphological analysis. The ratio of the total weight of all skeletal muscles on the right carcass side to the final BW was calculated (NY/T 825–2004, Ministry of Agriculture of the People’s Republic of China, 2004).

In Experiment 2, fourteen 28-day-old male piglets (Large White × Landrace) were housed individually in metabolism pens (1.5 × 0.75 m^2^) and assigned randomly to either of the two dietary groups as in Experiment 1, with 7 pigs/group. During the 4-week dietary treatment period, one pig from the control group was excluded because of sudden death due to unknown causes. Subsequently, all pigs (weighing ~20 kg) received surgery as previously described [24,27,28]. Briefly, after overnight feed deprivation, pigs were surgically implanted with catheters in the carotid artery (Tygon tubing, 2.41 mm OD), jugular vein (Central venous catheter, 14Ga, 20 cm), femoral artery and vein (Central venous catheter, 18 Ga, 20 cm) on the left side of the body, under isoflurane anesthesia and strict aseptic conditions. The catheters were filled with sterile saline containing heparin. The four blood vessels were tightly ligated to secure the catheters. Pigs received daily intramuscular injection of penicillin sodium (6000 IU/kg BW) and streptomycin sulfate (20 mg/kg BW) for 4 days. Five days after surgery, twelve of the thirteen pigs were in good health and with well-kept catheters, with six pigs for each group. The femoral artery catheter was used for the continuous infusion of p-amino hippurate (PAH) to measure plasma flow across hindlimb muscle. The jugular vein was used for infusion of NaH^13^CO_3_ and [1-^13^C]leucine to measure leucine metabolism in hindlimb muscle. Carotid artery and femoral vein catheters were used for blood collection.

### 2.2. Tracer Infusion and Blood Sampling Protocols

**Figure 1:**
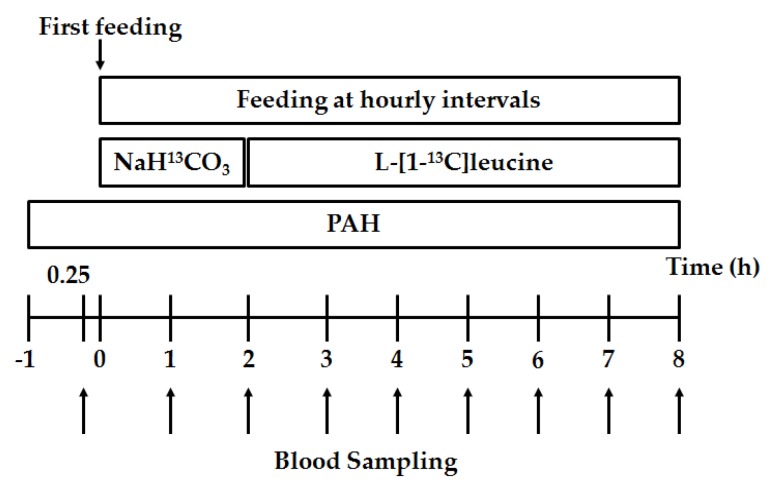
Outline of the tracer infusion and blood sampling protocols. After 15 min of blood sampling at baseline, pigs were fed at hourly intervals with intravenous infusion of NaH^13^CO_3_ for 2 h followed by a 6-h infusion of [1-^13^C]leucine. Blood samples were obtained every 1 h throughout the 8-h tracer infusion. One hour before the first feeding, an infusion of p-amino hippurate (PAH) was made and continued throughout the experimental period.

An overview of the infusion and blood sampling protocols is depicted in Figure 1. On Day 5 post surgery, pigs were provided with meals at hourly intervals after an overnight fast, and the meal was equivalent to 1/24 of the daily intake (45 g/kg BW), which was continued for 8 h to achieve a constant fed state [27,29]. Immediately after the starting of feeding, a primed (7.5 μmol/kg) and a continuous (10 μmol·kg^−1^·h^−1^) intravenous infusion of NaH^13^CO_3_ (Cambridge Isotope Laboratories, Andover, MA) were performed from 0 to 2 h to estimate CO_2_ production rate. Arterial and venous whole blood (5.0 mL) samples were obtained at baseline and every 1 h for the remaining 4 h for analyzing the steady-state CO_2_ production rate.

To quantify leucine kinetics in hindlimb muscle, a primed (10 μmol/kg) and a continuous (10 μmol·kg^−1^·h^−1^) intravenous infusion of [1-^13^C]leucine (Cambridge Isotope Laboratories) were performed from 2 to 8 h. Measurement of plasma flow across hindlimb muscle was performed by a primed (19.1 mL) and a continuous (0.788 mL/min) intra-arterial infusion of PAH throughout the experimental period. Arterial and venous blood samples (5.0 mL) were obtained at baseline and every 1 h throughout the 6-h leucine tracer infusion, and all the blood samples were immediately placed on ice. Then, the heparinized blood samples were centrifuged at 4 °C at 3000× *g* for 10 min and the supernatant fluid (plasma) was collected for analyzing the steady-state isotopic enrichment of [1-^13^C]leucine and [1-^13^C]KIC and concentrations of leucine, KIC and PAH.

### 2.3. Cross-Sectional Area Determination

The cross-sectional area (CSA) of LD muscle fibers was measured by classic hematoxylin and eosin staining as previously described by Zhang et al. [30]. Briefly, ten cross-sections per sample were stained with hematoxylin and eosin using standard paraffin embedding procedures, and the photographs were taken using an Olympus BX51 microscope (Olympus Optical Company, Tokyo, Japan). The fiber CSA of LD muscle was measured using Image-Pro Plus 6.0 image processing and analysis system. For each sample, 200–300 fibers were measured and the mean value was calculated.

### 2.4. Western Blotting

Protein expression of muscle samples was analyzed by western blotting as previously described [7]. Briefly, frozen LD muscle samples were powdered under liquid N and lysed in ice-cold RIPA buffer (50 mM Tris-HCl (pH 7.4), 150 mM NaCl, 1% Triton X-100, 1% sodium deoxycholate, 0.1% SDS, and 1 mM phenylmethylsulfonyl fluoride plus a 10 μL phosphatase inhibitors (P1260; Applygen Technologies Inc., Beijing, China)). Protein concentrations were determined using the BCA protein Assay Kit. Equal amounts of protein (30 μg) were separated on SDS-PAGE gels, transferred to polyvinylidene difluoride membranes (Millipore), and then were incubated with indicated primary antibodies. The horseradish peroxidase-linked secondary antibody was used at a 1:10,000 dilution in 1% BSA TBST. The chemiluminescence signal was detected by using the SuperSignal West Pico Chemiluminescent Substrate (Pierce, Rockford, IL, USA). Photographs of the membranes were taken using the Gel Logic Pro 2200 system (Carestream Molecular Imaging, Rochester, NY, USA) and quantified using Image J software.

### 2.5. Plasma Concentrations of Insulin, AA, PAH and KIC

Plasma insulin concentrations were measured using a porcine insulin RIA kit (Shanghai Biotechnologies Ltd., Shanghai, China) [5]. Free AA concentrations in plasma were determined by UHPLC-Q-Orbitrap HRMS method using AQC derivatization as described by Yin et al. [31]. Concentrations of PAH in plasma samples were detected spectrophotometrically on an automated analysis system (TEKAN, Infinite M200 Pro, Männedorf, Switzerland) [27]. Plasma KIC was o-phenylenediamine derivatized, ethyl acetate extracted, and quantified by LC-MS as previously described [32].

### 2.6. Analysis of Stable Isotope Tracer Enrichment

Blood samples for ^13^CO_2_ production were analyzed using isotope ratio mass spectrometry (IRMS) as previously described [33]. Briefly, an aliquot of whole blood (1.0 mL) was placed in a 10-mL vacutainer (Becton Dickinson, Franklin Lakes, NJ, USA) containing 1.0 mL of perchloric acid (10% wt/wt), gently mixed, and placed on ice for 60 s. Room air was filtered via a soda lime filter (Sodasorb, Grace Container Products, Lexington, MA, USA) to obtain air devoid of CO_2_. The air (approximately 8 mL) was injected into the 10-mL vacutainer containing 1:1 whole blood-perchloric acid. Between 8 and 10 mL of gas was withdrawn and transferred to a sterile 10-mL vacutainer for subsequent analysis of isotopic enrichment of ^13^CO_2_ via continuous flow GC-IRMS.

Isotopic enrichment of [1-^13^C]leucine and [1-^13^C]KIC was quantified by UHPLC-Q-Orbitrap HRMS method. Plasma sample preparation was performed according to Yin et al. [31] with modifications. Briefly, 300 μL plasma samples were extracted using 1.2 mL of ice-cold methanol. After vortexing, the samples were placed on ice for 30 min and centrifuged at 14,500 rpm for 10 min at 4 °C. The supernatants were collected and 500 μL aliquots of supernatants were evaporated to dryness in a vacuum concentrator. Residues were re-suspended in 100 μL of borate buffer, vortexed and centrifuged again at 14,500 rpm for 10 min at 4 °C. The supernatants passed through a 0.1 μm syringe filter were analyzed directly for the detection of [1-^13^C]KIC using UHPLC-Q-Orbitrap HRMS. The [1-^13^C]leucine in the supernatants was derivatized by adding derivatization reagent AQC dissolved in acetonitrile. Then, the solutions were vortexed and heated to 55 °C for 10 min. After cooling to room temperature, the solutions were transferred to sampler vials for UHPLC-Q-Orbitrap HRMS analysis. Standard solutions were derived in the same manner. The isotopic enrichment of [1-^13^C]leucine was determined in positive ionization mode by monitoring the mass-to-charge ratio of ions at 302/303, while the isotopic enrichment of [1-^13^C]KIC was determined in negative ionization mode by monitoring the mass-to-charge ratio of ions at 129/130.

### 2.7. Calculations

Calculations for plasma flow and leucine kinetics across hindlimb muscle are provided in the Appendix A.

### 2.8. Statistical Analysis

For all data analyses, an individual pig was used as an experimental unit. Data on skeletal muscle mass, CSA of LD muscle, plasma concentrations and isotopic enrichment of steady-state leucine and its metabolites, leucine kinetics and muscle protein expression were analyzed using Student’s t-tests. Pearson correlation coefficients were calculated among muscle net production of [1-^13^C]KIC and muscle protein turnover. All analyses were performed using the SAS 8.0. Data were expressed as mean ± SEM. A value of *p* < 0.05 was considered to be statistically significant.

## 3. Results

### 3.1. Skeletal Muscle Mass and Cross-Sectional Area of LD Muscle Fibers

In Experiment 1, twenty-three major individual skeletal muscles of the left carcass side were separated entirely and weighed. The results showed that dietary BCAA supplementation increased the mass of only supraspinatus muscle in the forequarter and almost all muscles in the hindquarter (*p* < 0.05) (Table 2). Meanwhile, the mass of LD muscle in the midquarter, which is the largest muscle in the body, was also increased by BCAA supplementation (*p* < 0.05). The mass of teres major, deltoids, psoas major, tensor fascia latae and adductor muscles was elevated in BCAA-supplemented group compared with in control group (*p* < 0.1). In the measurement of relative weight (%) of individual muscles, we found that only the gracilis muscle had a significantly higher relative weight in treatment group than in control group (0.288 ± 0.015 vs. 0.249 ± 0.008; *p* = 0.047). We further measured the total relative weight of all muscles on the right carcass side. The results showed that it was enhanced by BCAA supplementation (*p* < 0.05) (Table 2). Additionally, the CSA of LD muscle fibers was greater in BCAA-supplemented group than in control group (*p* < 0.05).

### 3.2. Plasma Concentrations and Isotopic Enrichment of Leucine and Its Metabolites

**Figure 2:**
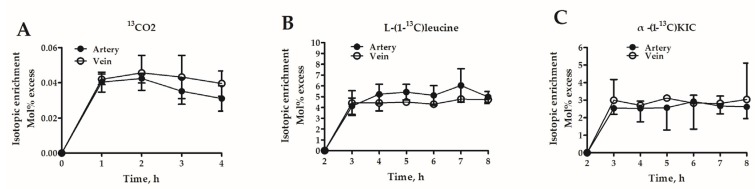
Time course of isotopic enrichment of: ^13^CO2 (**A**); [1-^13^C]leucine (**B**); and [1-^13^C]KIC (**C**) in carotid artery and femoral vein during intravenous infusion of NaH^13^CO_3_ and [1-^13^C]leucine (Experiment 2). Duration of NaH^13^CO_3_ infusion was 0–2 h with steady state reached between 1 and 2 h. Duration of [1-^13^C]leucine infusion was 2–8 h with steady state reached between 5 and 8 h for both [1-^13^C]leucine and [1-^13^C]KIC. Enrichment is expressed as mole percent excess. Values are means ± SEM (*n* = 3 pigs/group).

In Experiment 2, isotope-labeled tracers (i.e., [1-^13^C]leucine and NaH^13^CO_3_) and femoral vein plasma flow measurements were utilized to estimate leucine kinetics across hindlimb muscle in isotopic steady state (see APPENDIX). As shown in Figure 2A, steady-state of plasma isotopic enrichment (expressed as mole percent excess, MPE) of ^13^CO_2_ was achieved in artery and femoral vein after 1 h during the 2-h continuous infusion of NaH^13^CO_3_. Duration of [1-^13^C]leucine infusion was 2–8 h, and steady state for enrichment of both [1-^13^C]leucine and [1-^13^C]KIC was observed between 5 and 8 h (Figure 2B,C). Meanwhile, concentration of arterial leucine reached the peak within 5–8 h (139.69 ± 13.73 and 200.03 ± 29.86 μmol/L at 7 h for control and treatment, respectively) and was higher than that at baseline (113.70 ± 32.52 μmol/L and 124.38 ± 8.79 μmol/L for control and treatment, respectively). Therefore, steady-state concentrations and isotopic enrichment of leucine and KIC were estimated by the mean values of the samples collected at the last three time points. The samples collected at the 2nd time point were used to calculate the concentration and isotopic enrichment of CO_2_ at steady state.

The average concentrations and isotopic enrichment of steady-state leucine and its metabolites are presented in Table 3. In contrast to the control treatment, dietary BCAA supplementation increased the arterial concentrations of leucine and KIC and venous concentration of KIC. No differences in arterial and venous isotopic enrichment of leucine, KIC and CO_2_ as well as concentrations of arterial CO_2_ and venous leucine and CO_2_ were observed between two groups.

### 3.3. Leucine Kinetics across Hindlimb Muscle

Leucine kinetics of hindlimb muscle in Experiment 2 are shown in Table 4. Compared with control treatment, BCAA supplementation enhanced the arterial input and net uptake of leucine (*p* < 0.05), and thus the utilization of leucine by hindlimb muscle was significantly increased (*p* < 0.05). The majority of leucine utilized by hindlimb muscle was for protein synthesis, which was significantly greater in BCAA-supplemented group than in control group (*p* < 0.05). The remaining leucine was metabolized via transamination and decarboxylation. Of note, net transamination was elevated in response to BCAA supplementation (*p* < 0.05). However, no difference in leucine oxidation was observed between the two groups. Interestingly, along with the increase in protein synthesis, protein degradation was also increased after BCAA supplementation (*p* < 0.05). BCAA-supplemented group exhibited a significantly increased protein deposition (*p* < 0.05), which was in line with the increase of muscle mass.

### 3.4. Leucine Metabolic Fate in Hindlimb Muscle

**Figure 3:**
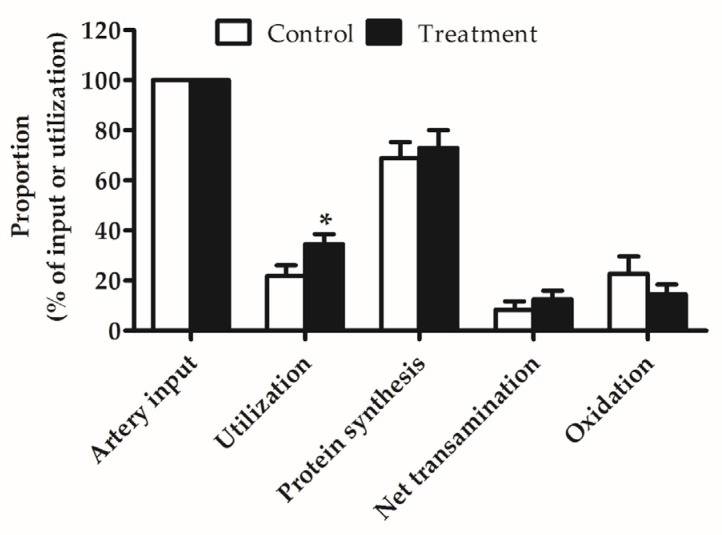
Fractional utilization (percent of artery input), protein synthesis, net transamination and oxidation (percent of utilization) of leucine in hindlimb muscle of piglets fed reduced-protein diets without (Control) or with (Treatment) supplemental branched-chain amino acids in the fed state (Experiment 2). Values are means ±SEM (*n* = 6 pigs/group). Trend toward statistical significance, * *p* = 0.06 versus control group.

As shown in Figure 3, the total net leucine utilized by hindlimb muscle only accounted for 22%–35% of the arterial leucine intake. In both groups, on average, approximately 70% of total leucine utilized by hindlimb muscle was retained for protein synthesis, and the remaining leucine was metabolized by hindlimb muscle via transamination and decarboxylation. Moreover, 10% of total leucine was used for production of KIC and 20% was oxidized to CO_2_ in hindlimb muscle. A higher proportion of input leucine was utilized in BCAA-supplemented group compared with in the control group (*p* = 0.06). No differences in fractional protein synthesis, net transamination and oxidation were detected between the two groups.

### 3.5. Muscle Net Uptake of [1-^13^C]Leucine and Production of [1-^13^C]KIC

**Figure 4:**
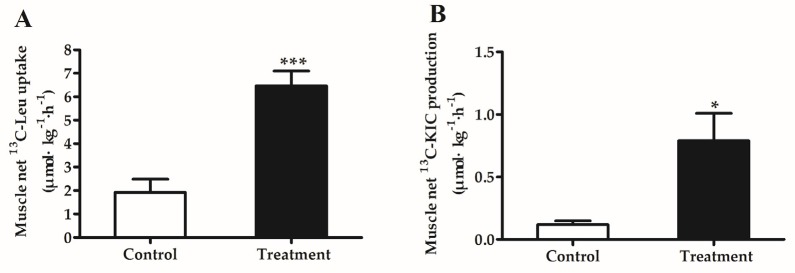
Muscle net [1-^13^C]leucine uptake (**A**); and [1-^13^C]KIC production (**B**) in piglets fed reduced-protein diets without (Control) or with (Treatment) supplemental branched-chain amino acids in the fed state (Experiment 2). Values are means ± SEM (*n* = 6 pigs/group). * *p* < 0.05 versus control group. *** *p* < 0.001 versus control group.

The first step of leucine catabolism is transamination, leading to the formation of KIC, and KIC may function as a potential regulator of muscle protein synthesis. Therefore, in Experiment 2, we detected the net [1-^13^C]leucine uptake and [1-^13^C]KIC production in hindlimb muscle. As shown in Figure 4, in line with the elevated muscle protein synthesis, the uptake of [1-^13^C]leucine and production of [1-^13^C]KIC were significantly increased by BCAA supplementation (*p* < 0.05).

### 3.6. Correlations

To further elucidate the potential role of KIC and muscle protein degradation in regulating muscle protein turnover, we calculated the correlations among these variables (Table 5). The uptake of ^13^C-leucine was strongly and positively correlated with ^13^C-KIC production, protein synthesis and degradation (*p* < 0.05). As expected, there was significant positive correlation between muscle protein synthesis and the production of [1-^13^C]KIC (*p* < 0.05). Significant positive correlation between protein synthesis and protein degradation was also observed (*p* < 0.05).

### 3.7. Protein Translation Initiation and Degradation Signaling of LD Muscle in the Fasted State

**Figure 5:**
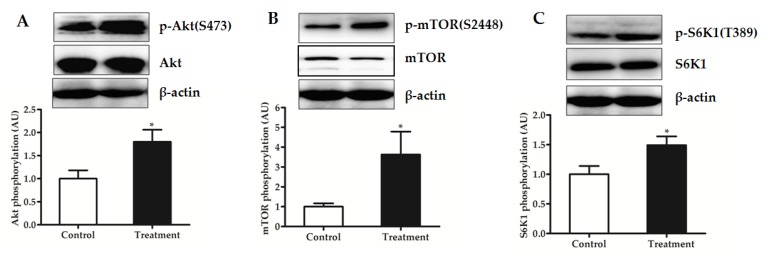
Phosphorylation of: Akt at Ser^473^ (**A**); mTOR at Ser^2448^ (**B**); and S6K1 at Thr^389^ (**C**) of longissimus dorsi muscle in piglets fed reduced-protein diets without (Control) or with (Treatment) supplemental branched-chain amino acids in the fasted state (Experiment 1). Relative total protein was used as an internal standard for normalization. Data are means ±SEM (*n* = 6 pigs/group). * *p* < 0.05 versus control group.

In Experiment 1, mTOR activity in LD muscle measured after an overnight fasting was affected by BCAA supplementation (Figure 5). Phosphorylation of mTOR (Figure 5B) and its target S6K1 (Figure 5C) was significantly higher in BCAA-supplemented group than in control group (*p* < 0.05). Meanwhile, phosphorylation of Akt, the key regulator upstream of mTOR pathway, was also increased by BCAA supplementation (*p* < 0.05) (Figure 5A). These data indicated an activation of Akt/mTOR signaling pathway in LD muscle in response to BCAA supplementation. However, plasma concentration (mU/L) of insulin did not differ between the control and treatment groups (35.3 ± 2.8 vs. 30.5 ± 3.6; *p* = 0.322).

**Figure 6:**
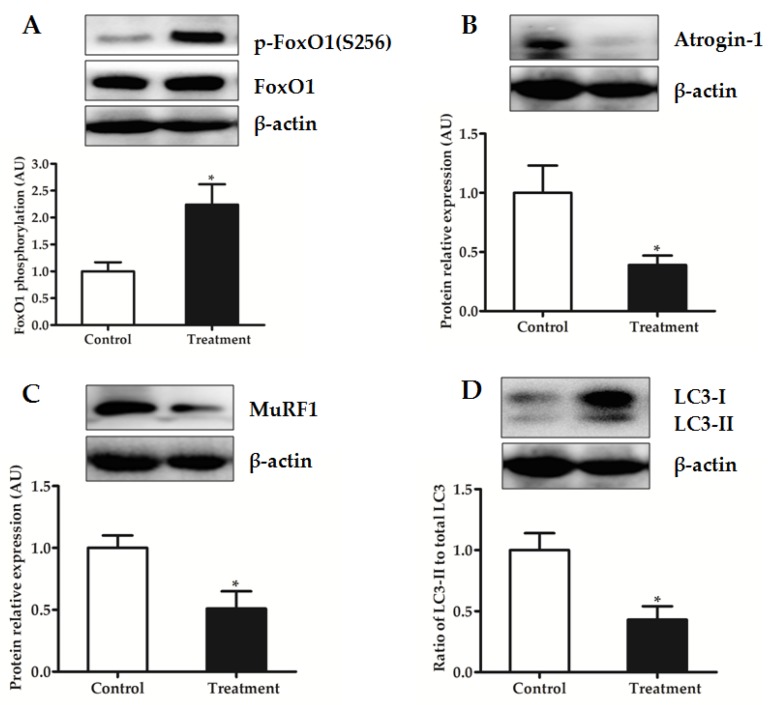
Phosphorylation of FoxO1 at Ser^256^ (**A**); relative protein abundances of atrogin-1 (**B**); and MuRF1 (**C**); and the ratio of LC3-II to total LC3 (**D**) of longissimus dorsi muscle in piglets fed reduced-protein diets without (Control) or with (Treatment) supplemental branched-chain amino acids in the fasted state (Experiment 1). Relative total protein or β-actin was used as an internal standard for normalization. Data are means ±SEM (*n* = 6 pigs/group). * *p* < 0.05 versus control group.

Moreover, after an overnight fasting, muscle phosphorylation of FoxO1, another important target of Akt, was higher in BCAA-supplemented group than in control group (*p* < 0.05) (Figure 6A). Correspondingly, relative protein levels of atrogin-1 (Figure 6B) and MuRF1 (Figure 6C), as well as the LC3-II to total LC3 ratio (Figure 6D), were decreased by BCAA supplementation (*p* < 0.05).

## 4. Discussion

The main objective of this study was to investigate the mechanisms involved in BCAA-induced increase of skeletal muscle mass. In the fed state, BCAA supplementation increased muscle protein deposition, which could be largely attributed to the significant increase in protein synthesis. We further found that the increase of protein synthesis by BCAA supplementation is associated with the enhanced protein degradation and KIC production from leucine catabolism in the muscle. Moreover, BCAA supplementation was found to enhance the fasted-state phosphorylation of protein translation initiation factors and inhibit the protein expression of proteolytic-related genes in both ubiquitin-proteasome and autophagy-lysosome systems.

### 4.1. BCAA Supplementation Increases Skeletal Muscle Mass in Piglets

Previous studies have suggested that individual skeletal muscles of pigs differ in protein turnover rates during postnatal growth and development [34,35]. Mulvaney et al. [35] showed that the protein accretion rate of the branchialis and semitendinosus muscles is consistent with the allometric growth of these muscles: semitendinosus muscle exhibits a later maturing, and higher allometric growth rate and protein accretion compared with branchialis muscle. Interestingly, Lefaucheur et al. [36] reported that a major effect of undernutrition is a reduction in the postnatal increase of myofiber CSA, which has a greater effect on longissimus lumborum muscle than on rhomboideus muscle in piglets. Of note, longissimus lumborum muscle has a higher postnatal allometric growth rate (1.10) than the earlier maturing rhomboideus muscle (0.84). We observed that the mass of most muscles in the hindquarter was significantly increased by BCAA supplementation, but the mass of nearly all of muscles in the forequarter was unaffected by the treatment (Table 2). The allometric growth rate of most muscles in the hindquarter, particularly in the hindlimb, was higher than that in the forequarter [37]. These results indicate that the muscles with high allometric growth rates may be sensitive to dietary nutrition changes, such as BCAA supplementation, which results in the increase of muscle mass in piglets.

Moreover, the mass of skeletal muscle is regulated by nutritional, hormonal and mechanical factors. Nutrients (especially AA) and hormones (especially insulin and IGF1) are essential for muscle growth in neonatal and young animals [38,39]. However, the maintenance/enhancement of adult skeletal muscle mass is primarily ensured/driven by mechanical factors (exercise and physical loading) [39,40]. Thus, adult skeletal muscle may not be as sensitive to nutrition changes as skeletal muscle during the rapid growth phase, and the mass may not be increased by BCAA supplementation.

### 4.2. BCAA Supplementation Increases Protein Synthesis, Protein Degradation and Conversion of Leucine to KIC in Skeletal Muscle of Piglets in the Fed State

The absorption of digested protein after feeding causes an elevation in plasma concentrations of AA, which results in an increased inward flux into muscle and thereby stimulates protein synthesis [12]. In this study, increase of muscle protein synthesis by BCAA supplementation was accompanied with the elevation of leucine input from plasma in the fed state (Table 4). Additionally, growing evidences indicate that coordinated regulation of protein synthesis and degradation has been recently reported in cells [13]. The possible physiological reasons for this response in cells may involve the maintenance of the intracellular pool of free AA through enhancing protein degradation to sustain the elevation of protein synthesis [13,41]. In contrast to the whole body protein degradation, muscle protein degradation during feeding in piglets is not reduced but rather is elevated [11]. This may also be confirmed by our result that BCAA supplementation increased muscle protein degradation in the fed state, which was strongly and positively correlated with muscle protein synthesis (Table 5). Therefore, an increase of protein degradation may be required for maintaining the intracellular concentration of free AA to supply sufficient substrates for protein synthesis in the muscle by BCAA supplementation in the fed state. Although both protein synthesis and degradation are stimulated by BCAA supplementation, the increase of protein synthesis is higher than that of protein degradation, leading to the increase of protein deposition and muscle mass.

Previous studies of isolated incubated muscle and adult humans suggested that diaphragm and forearm muscles can convert leucine to KIC and CO_2_ by transamination and decarboxylation, respectively [42,43]. As described above, muscles in the hindlimb of piglets are more sensitive to BCAA supplementation and exhibit increase in mass. We detected the quantitative metabolic fate of leucine and the association of leucine catabolism with protein synthesis in hindlimb muscle in vivo. Consistent with previous studies, this study in piglets using hindlimb flux model showed that approximately 30% of leucine extracted by hindlimb muscle is not used for protein synthesis, and is metabolized into KIC (40%) and CO_2_ (60%). The observation of significant transamination and decarboxylation in hindlimb muscle is supported by previous reports, which showed the presence of enzymes and their high activities involved in leucine metabolism in the semitendinosus muscle of pigs [44,45].

The high metabolization of leucine within skeletal muscle poses the possibility that metabolites of leucine are involved in mediating the anabolic effect of leucine on muscle growth. It has been shown that two major leucine metabolites KIC and HMB could stimulate muscle protein synthesis after oral administration or arterial infusion [21,22,46]. However, since the key enzyme that catalyzes KIC to produce HMB is absent in skeletal muscle [23], it seems unreasonable to attribute the increased muscle protein synthesis due to BCAA supplementation to elevated HMB production. Thus, we mainly detected the intramuscular production of KIC and investigated its potential role in regulating muscle protein synthesis. Indeed, in this study, muscle protein synthesis was significantly increased by BCAA supplementation, which is strongly and positively associated with the intramuscular production of KIC (Table 5). Furthermore, although infusion of KIC leads to elevated muscle protein synthesis along with the activation of translation initiation factors [21], it is unclear whether KIC itself or reversible transamination of KIC to leucine is required for this effect. Interestingly, in this study, increases of muscle net leucine extraction and KIC production were detected after BCAA supplementation. Meanwhile, the protein translation initiation signaling was also increased by BCAA supplementation. These results indicate that besides leucine, its metabolite KIC may also act as a signaling molecule to activate protein translation initiation, and partially mediate leucine dependent-stimulation of muscle protein synthesis.

### 4.3. BCAA Supplementation Promotes Protein Synthesis Signaling and Inhibits Degradation Signaling in Skeletal Muscle of Piglets in the Fasted State

Activation of mTOR results in the phosphorylation and activation of S6K1, which in turn phosphorylates ribosomal protein S6, and finally stimulates translation initiation and protein synthesis. In this study, phosphorylation of mTOR and S6K1 was significantly up-regulated by BCAA supplementation as expected (Figure 5). Previous studies have suggested that leucine modulates mTOR activity indirectly through PI3K/Akt signaling pathway in human skeletal muscle and cell models [46,47,48]. Indeed, we also observed an activation of the PI3K/Akt signaling pathway in the muscle. Thus, the increased muscle mass in response to BCAA supplementation may be due to, at least in part, the activation of translation initiation factors by increasing the activity of PI3K/Akt pathway. It seems unlikely that the action of BCAA is mediated by insulin, because the plasma level of insulin did not differ between the control and BCAA-supplemented groups.

Regulation of skeletal muscle mass depends on the balance between protein synthesis and degradation. In most types of muscle atrophy induced by various stimuli including fasting, the loss of mass is mainly attributed to the rapid increase in protein degradation [14,15]. However, there is limited evidence showing that leucine regulates protein degradation in muscle. Although muscle protein degradation is unaffected by ingestion of essential AA [49], other studies have shown a reduction in protein degradation after BCAA or leucine administration [16,50,51,52]. Previous studies have indicated that BCAA or leucine alone affects the ubiquitin-proteasome system through changing the mRNA levels of MAFbx/atrogin-1 and MuRF-1 during atrophic conditions [16,51,53], and influences the autophagy-lysosome system by regulating the expression of LC3II under physiological conditions [52,54]. In this study, BCAA supplementation inhibited the ubiquitin-proteasome system in the fasted state, as indicated by the increase in phosphorylation of FoxO1 and the reduction in protein levels of atrogin-1 and MuRF1 (Figure 6). Moreover, the decrease in the ratio of LC3-II to total LC3 indicates that BCAA administration also down-regulates the autophagy-lysosome system. Of note, Nakashima et al. [17] found that KIC administration after feed deprivation for 24 h could suppress the expression of proteolysis-related genes, and thus result in a decrease in myofibrillar proteolysis, while leucine is much less effective. Therefore, these results indicate that the intramuscular conversion of leucine to KIC may be required for the reduction of expression levels of proteins in both ubiquitin-proteasome and autophagy-lysosome systems by BCAA supplementation in the fasted state.

## 5. Conclusions

Supplementation of BCAA to reduced-protein diet increases the mass of skeletal muscles, particularly in the midquarter and hindquarter in a piglet model. We also provide in vivo kinetic evidence for leucine transamination and decarboxylation in the hindlimb muscle. Moreover, in the fed state, BCAA supplementation increases protein degradation and production of KIC from leucine, both of which could partially contribute to the BCAA induced-stimulation of protein synthesis in skeletal muscle. In the fasted state, BCAA supplementation suppresses the expression of proteolysis-related genes, which may also be mediated by KIC production. These results provide a direct biochemical explanation for the anabolic effect of BCAA supplementation on muscle mass. Our findings also highlight the physiological significance of the conversion of leucine to KIC in regulating muscle protein synthesis and degradation.

## Figures and Tables

**Table 1:** Ingredients and nutrient contents of experimental diets.

Items	Control	Treatment
Ingredient (%)		
Corn	70.09	70.09
Soybean meal	10.70	10.40
Whey powder	5.00	5.00
Fish meal	4.00	4.00
Concentrated soybean protein	5.00	5.00
Soybean oil	0.40	0.50
l-Lys·HCl	0.48	0.48
DL-Met	0.23	0.23
l-Thr	0.22	0.23
l-Trp	0.06	0.06
l-Ile	-	0.17
l-Leu	-	0.24
l-Val	-	0.16
l-Ala	0.42	-
Dicalcium phosphate	1.30	1.30
Limestone	0.60	0.60
Salt	0.30	0.30
Bentonite	0.20	0.24
Premix *	1.00	1.00
Analyzed composition (%)		
CP	16.7	16.7
Ether extract	8.48	8.95
Crude Fiber	2.34	2.54
Lys	1.32	1.34
Met + Cys	0.88	0.88
Thr	0.87	0.92
Trp	0.21	0.22
Ile	0.60	0.81
Leu	1.45	1.83
Val	0.76	0.95
His	0.74	0.77
Phe	0.42	0.44
Arg	1.02	1.02
Calculated composition †		
NE, MJ/kg	10.38	10.38
SID (Met + Cys):Lys	0.55	0.55
SID Thr:Lys	0.59	0.59
SID Trp:Lys	0.16	0.16
SID Ile:Lys	0.47	0.61
SID Leu:Lys	1.07	1.27
SID Val:Lys	0.55	0.68

* Provided per kilogram of diet (as-fed basis): vitamin A, 15000 IU; vitamin D3, 1500 IU; vitamin E, 30 mg; vitamin B1, 3.2 mg; vitamin B2, 8 mg; vitamin B6, 4 mg; vitamin B12, 0.03 mg; vitamin K3, 3.2 mg; niacin, 34 mg; folate, 1.6 mg; pantothenic acid, 18 mg; biotin, 0.2 mg; choline chloride, 500 mg; Cu, 150 mg; Fe, 120 mg; Mn, 45 mg; Zn, 90 mg; I, 0.6 mg; Se, 0.3 mg; Co, 0.3 mg; chlortetracycline, 35 mg; tiamulin 12 mg. † Values for standardized ileal digestible (SID) amino acids and net energy (NE) were calculated according to NRC [25].

**Table 2:** Skeletal muscle mass and cross-sectional area of longissimus dorsi muscle in piglets fed reduced-protein diets without (Control) or with (Treatment) supplemental branched-chain amino acids (Experiment 1).

Items	Control	Treatment	Pooled SEM	*p* Value
Muscle mass in the forequarter (g)				
Trapezius	25.0	29.2	5.5	0.469
Supraspinatus	78.3	105.8	9.0	0.012
Infraspinatus	55.0	57.5	6.4	0.705
Teres major	23.3	27.5	2.0	0.065
Deltoids	14.2	16.7	1.3	0.092
Subscapularis	22.5	26.7	2.4	0.111
Tricepsbrachii	148.3	170.8	13.0	0.115
Tensor fasciae antebrachii	10.8	11.7	2.3	0.721
Biceps	36.7	42.5	3.7	0.150
Brachii	23.3	25.0	1.7	0.341
Muscle mass in the midquarter (g)				
Latissimus dorsi	90.0	99.2	10.6	0.407
Pectoralis profundus	109.0	116.6	6.8	0.272
Longissimus dorsi	401.7	517.5	43.6	0.024
Psoas major	54.2	61.7	4.1	0.097
Muscle mass in the hindquarter (g)				
Glutaeus superficialis	120.8	150.0	9.0	0.009
Gluteus medius	25.8	34.2	3.4	0.033
Biceps femoris	245.0	335.0	33.4	0.023
Semitendinosus	64.2	85.8	7.3	0.014
Semembranosus	202.5	239.2	12.4	0.014
Tensor fascia latae	36.7	44.2	3.7	0.068
Gracilis	43.3	60.8	4.1	0.002
Adductor	67.5	80.8	6.2	0.058
Quadriceps femoris	187.5	240.8	14.2	0.004
Relative total muscle mass (%) *	20.07	21.24	0.44	0.025
Cross-sectional areas (μm^2^)				
Longissimus dorsi	784	1086	95	0.010

Data are means and pooled SEM (*n* = 6 pigs/group). Differences were considered significant at *p* < 0.05. * All skeletal muscles on the right side of carcass were totally separated and weighed, and values are expressed as percentages relative to the final body weight.

**Table 3:** Steady-state leucine, α-ketoisocaproate and CO_2_ concentrations and isotopic enrichment in plasma of piglets fed reduced-protein diets without (Control) or with (Treatment) supplemental branched-chain amino acids in the fed state (Experiment 2).

Items	Control	Treatment	Pooled SEM	*p* Value
Arterial leucine				
Concentration, μmol/L	135.80	187.81	21.63	0.037
[1-^13^C]leucine, MPE	4.68	6.41	1.05	0.130
Venous leucine				
Concentration, μmol/L	124.67	154.30	18.82	0.146
[1-^13^C]leucine, MPE	3.94	4.97	0.61	0.121
Arterial KIC				
Concentration, μmol/L	46.84	64.88	4.77	0.004
[1-^13^C]KIC, MPE	1.43	2.10	0.53	0.238
Venous KIC				
Concentration, μmol/L	47.86	72.42	6.28	0.003
[1-^13^C]KIC, MPE	1.58	2.46	0.54	0.133
Arterial CO_2_				
Concentration, mmol/L	18.64	18.62	1.07	0.988
^13^CO_2_, MPE	0.0290	0.0333	0.0056	0.453
Venous CO_2_				
Concentration, mmol/L	22.98	20.70	1.47	0.153
13CO_2_, MPE	0.0259	0.0329	0.0060	0.271

Values are means and pooled SEM (*n* = 6 pigs/group). Differences were considered significant at *p* < 0.05. KIC, α- ketoisocaproate. MPE, mole percent excess.

**Table 4:** Leucine kinetics in hindlimb muscle of piglets fed reduced-protein diets without (Control) or with (Treatment) supplemental branched-chain amino acids in the fed state (Experiment 2).

Items	Control	Treatment	Pooled SEM	*p* Value
Arterial input	176.52	329.01	53.28	0.017
Net uptake	14.90	57.35	10.92	0.003
Utilization	38.60	106.26	14.57	0.001
Net transamination	2.44	14.38	5.21	0.045
Oxidation	8.96	17.06	7.47	0.304
Protein synthesis	27.21	74.82	10.92	0.001
Protein degradation	23.71	48.91	11.21	0.048
Protein deposition	3.50	25.91	8.51	0.025

Values are means and pooled SEM (*n* = 6 pigs/group). Leucine kinetics are expressed as μmol·kg^−1^·h^−1^. Differences were considered significant at *p* < 0.05.

**Table 5:** Correlation coefficients among muscle net ^13^C-leucine uptake, ^13^C-KIC production and muscle protein turnover of piglets in the fed state (Experiment 2).

	^13^C-Leucine Uptake	^13^C-KIC Production	Protein Synthesis	Protein Degradation	Protein Deposition
^13^C-leucine uptake	1.00	0.70 *	0.86 **	0.73 *	0.43
^13^C-KIC production		1.00	0.64 *	0.55	0.31
Protein synthesis			1.00	0.83 **	0.54
Protein degradation				1.00	−0.02
Protein deposition					1.00

Differences were considered significant at: * *p* < 0.05, ** *p* < 0.01. KIC, α-ketoisocaproate.

## Abbreviations

The following abbreviations are used in this manuscript:

AA	Amino acids
BCAA	Branched-chain AA
KIC	α-ketoisocaproate
HMB	β-hydroxy-β-methylbutyrate
CP	Crude protein
SID	Standardized ileal digestible
LD	Longissimus dorsi
BW	Body weights
PAH	*p*-amino hippurate
CSA	Cross-sectional areas
IRMS	Isotope ratio mass spectrometry
MPE	Mole percent excess
mTOR	Mammalian target of rapamycin
S6K1	Ribosomal protein S6 kinase 1
Akt	Protein kinase B
LC3	Microtubule-associated protein 1 light chain 3
NE	Net energy

## Appendix A

Plasma flow across the hindlimb muscle was estimated using the PAH indicator dilution technique, and was calculated using the following Equation:

(A1)
MPF = C_i_ × IR × [(PAH_v_ − PAH_a_) × BW]^−1^

where MPF is muscle plasma flow (L·kg^−1^·h^−1^); C_i_ is PAH concentration in infusion solution (g/L); IR is infusion rate (L/h); PAH_v_ and PAH_a_ are concentrations (g/L) of PAH detected in the femoral vein and carotid artery, respectively; and BW is body weight (kg).

Leucine kinetics (μmol·kg^−1^·h^−1^) across the hindlimb muscle were determined by the following standardized equations:

(A2)
Arterial leucine input = C_a1_ × MPF

(A3)
Venous leucine output = C_v1_ × MPF

(A4)
Net leucine uptake = Equation (A2) − Equation (A3)

(A5)
Net leucine utilization = [(C_a1_ × E_a1_ − C_v1_ × E_v1_)]/(C_a1_ × E_a1_) × Equation (A2)

(A6)
Leucine release from protein degradation = Equation (A3) − (Equation (A2) − Equation (A5))

(A7)
Net leucine transamination = [(C_v2_ × E_v2_ − C_a2_ × E_a2_)]/(C_a1_ × E_a1_) × Equation (A2)

(A8)
Leucine oxidation = [(C_v3_ × E_v3_ − C_a3_ × E_a3_)]/(C_a1_ × E_a1_) × Equation (A2)

(A9)
Leucine used for protein synthesis = Equation (A5) − Equation (A7) − Equation (A8)

(A10)
Net protein balance (protein deposition) = Equation (A9) − Equation (A6)

(A11)
Net uptake of ^13^C-leucine = (C_a1_ × E_a1_ − C_v1_ × E_v1_) × MPF

(A12)
Net production of ^13^C-KIC = (C_v2_ × E_v2_ − C_a2_ × E_a2_) × MPF

where C_a1_ is arterial leucine concentration, C_v1_ is venous leucine concentration, C_a2_ is arterial KIC concentration, C_v2_ is venous KIC concentration, C_a3_ is arterial CO_2_ gas concentration, C_v3_ is venous CO_2_ gas concentration, E_a1_ is isotopic enrichment of arterial [1-^13^C]leucine, E_v1_ is isotopic enrichment of venous [1-^13^C]leucine, E_a2_ is isotopic enrichment of arterial [1-^13^C]KIC, E_v2_ is isotopic enrichment of venous [1-^13^C]KIC, E_a3_ is isotopic enrichment of arterial ^13^CO_2_, E_v3_ is isotopic enrichment of venous ^13^CO_2_, and MPF is muscle plasma flow. Concentrations of leucine, KIC and CO_2_ are expressed as μmol/L. Isotopic enrichment is expressed as mole percent excess. Plasma flow is expressed as L·kg^−1^·h^−1^.
